# Genetic association of *FTO/IRX* region with obesity and overweight in the Polish population

**DOI:** 10.1371/journal.pone.0180295

**Published:** 2017-06-29

**Authors:** Marta Sobalska-Kwapis, Aleksandra Suchanecka, Marcin Słomka, Anna Siewierska-Górska, Ewa Kępka, Dominik Strapagiel

**Affiliations:** 1 Biobank Lab, Department of Molecular Biophysics, University of Lodz, Lodz, Poland; 2 BBMRI.pl Consortium, Lodz, Poland; 3 Department of Molecular Biophysics, University of Lodz, Lodz, Poland; Garvan Institute of Medical Research, AUSTRALIA

## Abstract

**Background/Objectives:**

Genome-wide association studies (GWAS) have identified many *loci* associated with body mass index (BMI) in many different populations. Variants in the *FTO locus* are reported to be one of the strongest genetic predictors of obesity. Recent publications pointed also to a topologically associated domain (TAD) which is identified as a novel region affecting BMI. The TAD area encompasses the IRXB cluster (*IRX3*, *IRX5*, *IRX6*), *FTO* and *RPGRIP1L* genes.

**Subjects/Methods:**

In this study, we investigated the relationship between variation of the *FTO* and *IRX* genes and obesity in Poles. We presented a case—control association analysis (normal versus overweight and/or obesity group) of Polish adult individuals (N = 5418). We determined whether or not the chromosomal region 16:53 500 000–55 500 000 contains polymorphic variants which are correlated with BMI in Polish population, including sex and age stratified analysis.

**Results:**

The obtained results showed that the problem of weight-height abnormalities differently affects populations of Polish women and men (χ^2^ = 187.1; p<0.0001). From 353 SNPs enrolled to this study, 86 were statistically significant (highest χ^2^ = 15.72; p = 7.35E-05 observed for rs1558902). Linkage disequilibrium (LD) analysis revealed 61 blocks in the tested region of chromosome 16, with 24 SNPs located within the same block (block 8) of approximately 40 kb, in almost complete LD (|D’|>0.98, r^2^>0.80). We confirmed presence of the genetic susceptibility *loci* located in intron 1 of the *FTO* gene, which were correlated with BMI in our study group. For the first time, our analyses revealed strong association of *FTO* intronic variants (block 8) with overweight in group of men only. We have also identified association of the *IRX* region with overweight and/or obesity in Polish individuals.

**Conclusion:**

Our study demonstrated how tested SNPs make differential contributions to obesity and overweight risk. We revealed sex dependent differences in the distribution of tested *loci* which are associated with BMI in the population of Poles.

## Introduction

Obesity is one of the biggest health care problems worldwide. It is the most common nutritional disorder in developed countries, and it increases risk for hypertension, cardiovascular disease, and type 2 diabetes [1, 2]. It is a complex, multifactorial medical condition, affected by genetic and environmental risk factors [3–5]. According to the World Health Organization (2014) there are around 2 billion overweight adults, of those 670 million are considered to be affected by obesity (BMI ≥30 kg/m^2^) and 98 million severely affected by obesity (BMI ≥35 kg/m^2^). In Poland the current prevalence of overweight and obesity among the Polish population have been estimated to 36.54 and 13.34%, respectively (*WHO website* http://www.who.int/bmi*)*.

Twin and adoption studies have revealed that genetic factors contribute to 40–70% variation of BMI within a population [6, 7]. Large-scale GWAS have identified many *loci* associated with BMI. In 2007 three independent studies identified *FTO* as obesity susceptibility gene [8–10]. A number of different obesity-susceptible *loci* have been reported by the GWAS approach (*MC4R*, *MC3R*, *SLC6A14*, *TMEM18*, *POMC*, *BDNF*, *NEGR1*) [11]. Among these findings, the biggest effect on obesity phenotype till date has the *FTO*. The *FTO* gene is located on the long arm of chromosome 16 (16q12.2) and encodes a protein with the activity of 2-oxoglutarate- and iron-dependent nucleic acid demethylase (II). The *FTO* is expressed in many tissues including hypothalamus, pituitary and adrenal glands [8, 12].

The BMI influencing variants of the *FTO* are associated with increased intake of energy [13], dietary fat [14], protein [15], increased appetite and reduced satiety [16] and loss of control over eating [17]. Fully functional *FTO* is critical for normal development and physiology [18]. Impact of obesity predisposing variants of *FTO* on BMI changes throughout life. They do not influence birth weight [19]; body weight starts to be affected in early childhood, and declines through adulthood [20–23]. Sex influence obesity through hormonal, genetic and environmental factors [24, 25]. This reflects in sex-specific body fat distribution and is associated with it genetic *loci* [26].

Numerous studies confirmed the association between *FTO* common variants and obesity phenotype in many different populations [9, 10, 27]. Up to date, the genetic variability of *FTO* in the Polish population was not explored well. There are few studies which focused only on distribution of rs9939609 AA [22, 23, 28] variant and its association with higher BMI in Polish children and adults. Wrzostek et al. found the association of the G allele of rs9930506 with higher BMI among Polish adult subjects [29]. Multiple studies have also focused on deciphering potential mechanisms, by which variants within a region of high LD in introns 1 and 2 of *FTO* confer the obesity risk [30]. Recent evidence from Landgraf and colleagues suggested that *FTO* variants directly affect adipocyte function through targeting *IRX3* and *IRX5* [30]. Presence of the *FTO* risk haplotype in lean children was found to be associated with increased adipocyte-specific expression of *IRX3* and *IRX5*, whereas it was unaffected in a group of obese children.

The objective of the present study is to determine (by means of case-control association analysis) in what manner the polymorphic risk variants in FTO/IRXB region affect obesity and/or overweight in the Polish population. This is the first comprehensive study of cross-sectional individuals from the population at large, with representation from each geographical region of Poland. Presented case-control analysis comprising men and women subgroups gave a novel point of view on genetic association of *FTO* and IRXB cluster with obesity and overweight which depend on sex and age.

## Materials and methods

### Description of the participants

The participants were recruited in years 2010–2012 within research project TESTOPLEK and registered as a POPULOUS collection at the Biobank Lab of The Department of Molecular Biophysics of The University of Lodz [31]. Sampling was performed by a professional public opinion polling and survey-taking company (SMG/KRC Poland, a Millward Brown subsidiary). Each subject gave the written informed consent and fulfilled questionnaire. The saliva was collected into Oragene OG-500 DNA collection/storage receptacles (DNA Genotek, Kanata, Canada) from each individual. The approval for this study was obtained from The University of Lodz’s Review Board (KBBN-UL/II/2014). All procedures were performed in accordance with the Declaration of Helsinki (ethical principles for medical research involving human subjects).

**Fig 1:**
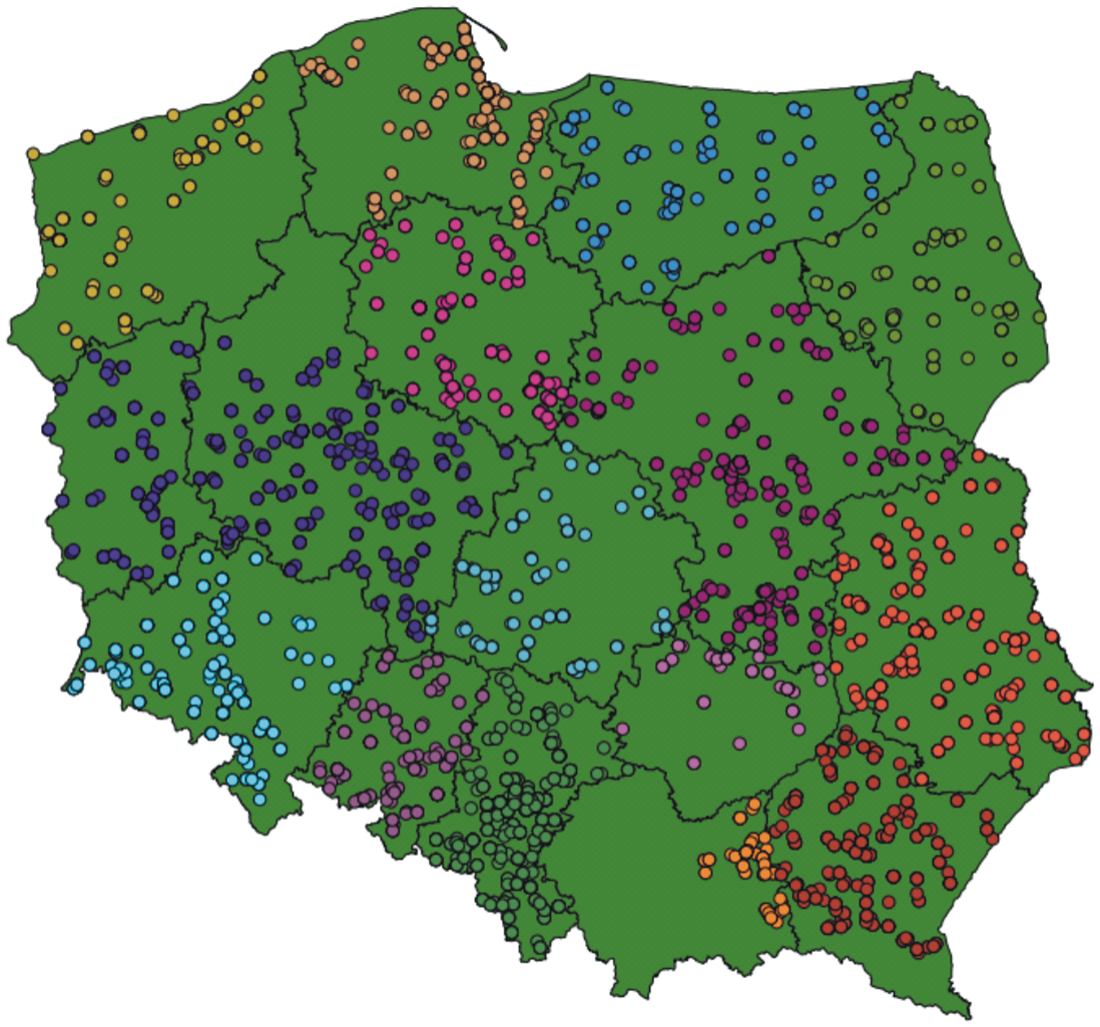
Geographic residence place of recruited participants included in study group.

From over 10000 individuals throughout Poland, a total of 6047 participants were involved in creation a study group (Fig 1). The exclusion criteria were: diabetes, leukemia, bone marrow transplantation and cancer. Individuals who declared any of these diseases were excluded from the study (n = 488). A total of 5559 subjects declared themselves healthy. The study group consisted of 2812 females and 2747 males.

BMI of each participant was calculated from the standard formula: BMI = weight/height^2^, based on self-assessment of height and weight controlled by trained pollsters.

### DNA extraction and quantification

The saliva samples were stored in room temperature until initial processing. DNA was manually isolated from 500 μl of saliva using the manufacturer procedure (PrepitL2P, PD-PR-052, DNA Genotek, Kanata, Canada). The elution volume was 50 μl. DNA was quantified using broad range Quant-iT^™^ dsDNA Broad Range Assay Kit (Invitrogen^™^, Carlsbad, CA, USA). All DNA samples underwent PCR reaction for sex verification, which is a standard procedure of DNA quality control in our laboratory [32]. Only the DNA samples with proper concentration and quality were included and were diluted to 50 ng/μl in sterile DNase free water. The concentration was checked again using the same fluorometric method.

All laboratory methods related to the samples management were performed according to the standard operating procedures (SOPs) of Biobank Lab.

### Genotyping

A total of 5559 DNA samples were genotyped for >550 000 SNPs using the 24x1 Infinium HTS Human Core Exome (Illumina Inc., San Diego, CA, USA) according to the protocol provided by the manufacturer. Briefly, DNA samples were amplified, then enzymatically fragmented and hybridized to the BeadChips. Afterwards, the BeadChips underwent an extension and X-staining processes. Next the BeadChips were scanned using iScan (Illumina Inc., San Diego, CA, USA). Raw fluorescence intensities were imported to the GenomeStudio with the Genotyping Module. All the data first underwent stringent quality control including sample exclusion (n = 141) if call rate was below 0.94 and 10% GenCall parameter was below 0.4. We filtered the data from the *FTO* and *IRX* region (NC_000016.9 (53 500 000–55 500 000)) which gave 353 SNPs for the analysis. All the SNPs reported in the presented study passed visual inspection for cluster quality. The results were exported from GenomeStudio using PLINK Input Report Plug-in v2.1.3 by forward strand. All sequence coordinates in this study are followed by GRCh37/hg19 reference sequence and were obtained from GenBank (http://www.ncbi.nlm.nih.gov).

### Statistical analysis

Associations between SNPs and normal weight (control group) versus overweight and/or obesity (case group) were carried out using PLINK 2.050 [33] (http://pngu.mgh.harvard.edu/purcell/plink/).

All of the tested SNPs were first analyzed for consistency with the exact test for Hardy—Weinberg Equilibrium implemented by Wigginton et al. [34]. Differences in the distributions of allele frequencies between normal weight vs. overweight, normal weight vs. obese and normal weight vs. overweight & obese groups were evaluated under additive, dominant, and recessive inheritance models adjusted for sex and age. For these, significantly correlated with BMI categories, the odds ratio (OR) with 95% confidence interval (CI) was estimated. We have also applied the Cochran—Mantel—Haenszel (CMH) statistics to test association of SNPs with overweight and/or when assuming age stratification in group of female and male [35, 36].

Moreover, to investigate the impact of the tested polymorphisms on BMI, we have also performed a linear regression analysis, with BMI as a continuous trait and age and sex as covariates. Analysis of LD and calculation of haplotype frequencies were performed using the Haploview 4.2 software [37] (http://www.broadinstitute.org/haploview/haploview). Associations of estimated haplotypes were carried out using the case-control allelic chi-squared test.

## Results

### Characteristic of the study group

A total of 5559 unrelated Polish adult subjects were enrolled to the study group. According to the GWAS exclusion criteria described above we excluded 141 samples due to bad quality of the genotyping. After exclusion, there were 2758 women and 2660 men (N = 5418). Case (obesity and/or overweight individuals) and control (normal weight individuals) groups were established from the study group according to the BMI (kg/m^2^). The sample size for obese subjects (BMI ≥ 30 kg/m^2^) was 797 (male:female ratio 424:373, age 50.16 ± 13.55 years, BMI 33.15 ± 3.33 kg/m^2^), whereas for normal weight controls (BMI 18.5–24.99 kg/m^2^) was 2624 (male:female ratio 1127:1497, age 38.74 ± 14.13 years, BMI 22.27 ± 1.71 kg/m^2^). The underweight (BMI < 18.5 kg/m^2^) and overweight (BMI 25–29.99 kg/m^2^) groups consisting of 192 (male:female ratio 155:37, mean age 32.30 ± 12.31 years, BMI 17.49 ± 0.92 kg/m^2^) and 1805 individuals (male:female ratio 1072:733, with mean age 45.48 ± 14.25 years and BMI 27.19 ± 1.39 kg/m^2^), respectively, were also included to the analysis (Table 1).

**Table 1:** Characterization of the study group (N = 5418).

Group	BMI [kg/m^2^]	N = 5418	Male:Female Ratio	Mean BMI [kg/m^2^]	Mean Age [years]
UNDERWEIGHT	<18.5	192	155: 37	17.5 ± 0.9	32.3 ± 12.3
NORMAL WEIGHT	18.5–24.99	2624	1127: 1497	22.3 ± 1.7	38.7 ± 14.1
OVERWEIGHT	25–29.99	1805	1072: 733	27.2 ± 1.4	45.5 ± 14.3
OBESITY	≥30	797	424: 373	33.1 ± 3.3	50.2 ± 13.6

Mean BMI ±SD; Mean Age ±SD

In our study we successfully genotyped SNPs spanning the 2 Mb region, encompassing the *RPGRIP1L*, *FTO*, *IRX3*, *IRX5*, *IRX6* genes. This region was selected according to the Hunt et al. [38] to encompass a TAD defined in embryonic stem cells [39]. We found 353 SNPs in the tested area. We estimated the minor allele frequency (MAF) of all mentioned SNPs and realized that 89 of them had a MAF under 0.01 (S1 Table) so we excluded them from the further analysis. In the case and control groups (S2 Table), two SNPs, rs4784382 and rs1211435, showed evidence for deviations from Hardy-Weinberg equilibrium (HWE; p<1E-04) and they were also excluded. Total genotyping rate in all individuals was 0.999 for all tested SNPs.

In summary, we conducted association analyses for 262 SNPs with an estimated MAFs > 1% in 5418 unrelated Polish adults, divided into four groups as described above and presented in Table 1. The ratio of women to men in our study group was 1.037 (50.9%: 49.1%).

### Linkage disequilibrium and haplotypes association analysis

**Fig 2:**
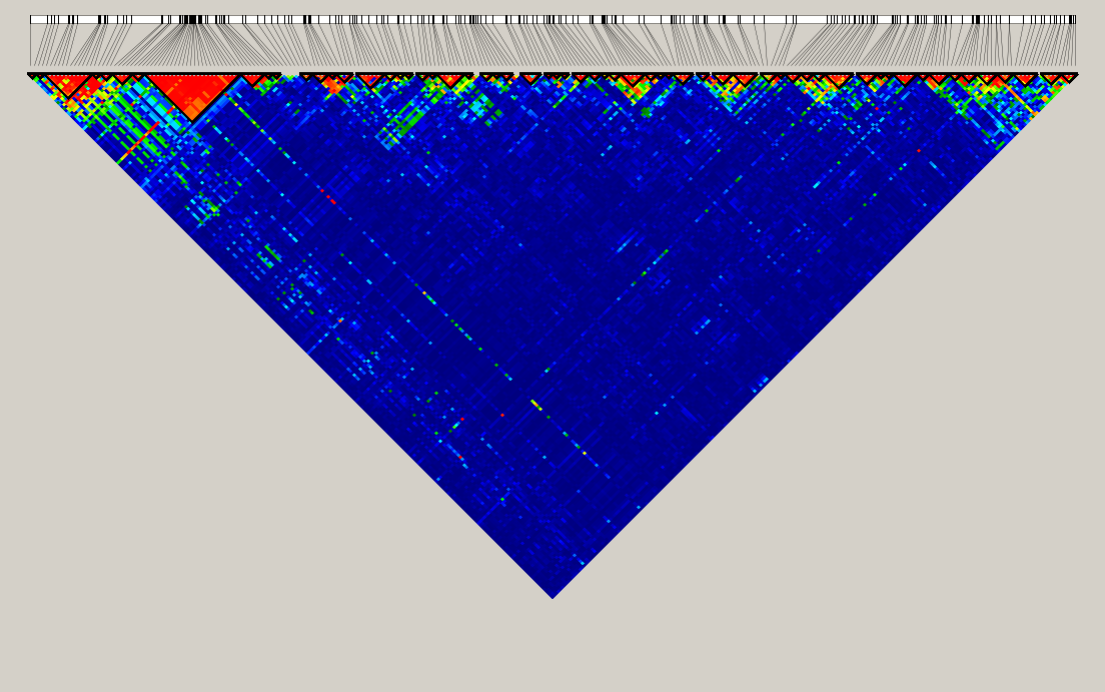
Linkage disequilibrium analysis of 262 SNPs from chromosomal region 16:53 500 000–55 500 000. Pairwise linkage exhibited as LD coefficients (|D’|) between each pair of SNPs. Minor allele frequencies of all SNPs used in this analysis are ≥10%. Bolded triangles show haplotype blocks. Data were generated with Haploview version 4.2.

We investigated the role of haplotypes formed by tested SNPs (n = 262) in the risk for obesity in our study group. Haplotyping was performed with Haploview [37]. As shown in Fig 2, LD analysis revealed 61 blocks in the tested region of chromosome 16, with 24 SNPs located within the same block 8 of approximately 42 kb, in almost complete LD (|D’|>0.98). This previously identified region in the first intron of the *FTO* contains rs76804286, localized next to the rs9939609, which is in complete dependence with all the SNPs in this block and with six SNPs from the block 3, and also with SNPs from the blocks 4, 5, 6 and 7. Haplotypes are depicted at S1 Fig. The strongest obesity associating haplotype (χ^2^ = 11.474, p = 0.0007) was in block 8 and occurred at a frequency of 0.46 in the case group and 0.41 in controls (S3 Table). Two other haplotypes were also associated with higher BMI: first in block 30 which is localized 200 kb upstream *IRX3* and second in 57 localized 8 kb upstream *IRX6*, (p = 0.0079 and 0.0097, respectively).

### Case-control association studies

Using 262 selected SNPs, we performed tests of independence between BMI and alleles of each SNP using differently coded control and case phenotype group: normal vs. overweight, normal vs. obesity, normal vs. overweight and obesity. As the underweight group was represented by only 192 individuals we excluded it from association analysis.

We found that the association of alleles of tested SNPs with BMI was different in group of women and men (S4–S6 Tables). The most interesting finding from our analysis is that the *FTO* intronic region (47 kb block 8) was strongly associated with overweight only in males without impact of SNPs from this block on overweight in a group of females. Surprisingly, analysis of obesity phenotype in sex stratified manner did not revealed sex differences in this region (S2 and S3 Figs, respectively). A case-control analysis for obese female and male groups without sex stratification was performed and results are presented in supplementary materials (S7 Table and S4 Fig).

**Fig 3:**
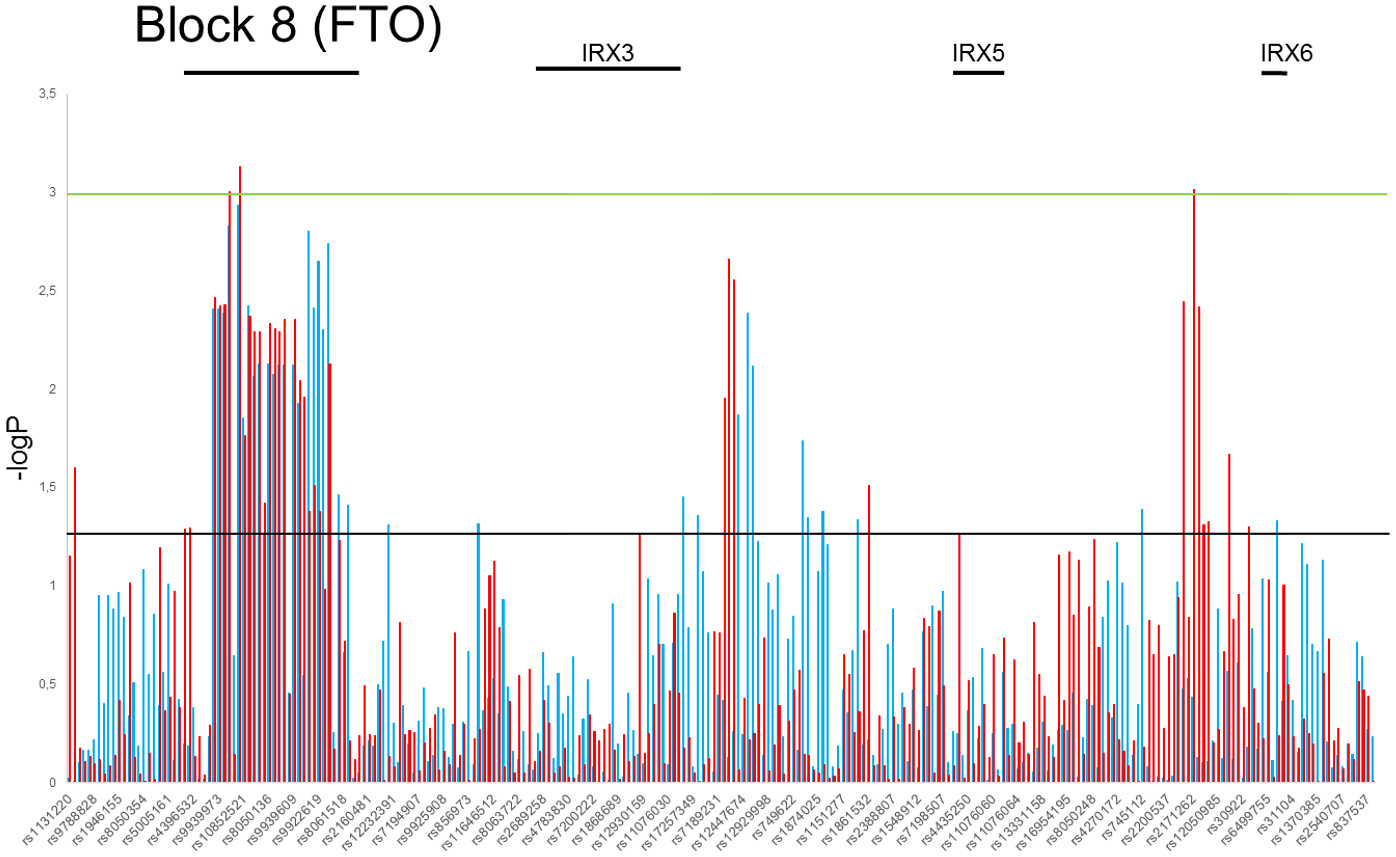
Distribution of estimated p values of the Chi square test in obesity phenotype stratified by age and sex. P values were calculated in the test of independence between the obesity phenotype and MAFs at tested SNPs (S4 Table). The *y* axis shows the −log_10_ p values of 262 SNPs obtained in obesity versus normal weight association analysis, and the *x* axis shows their rs numbers. Horizontal black and green lines represent the thresholds of p = 0.05 for significance without multiple correction and p = 8.2E-04 for LD block significance, respectively. Red and blue columns represents females and males group, respectively.

**Fig 4:**
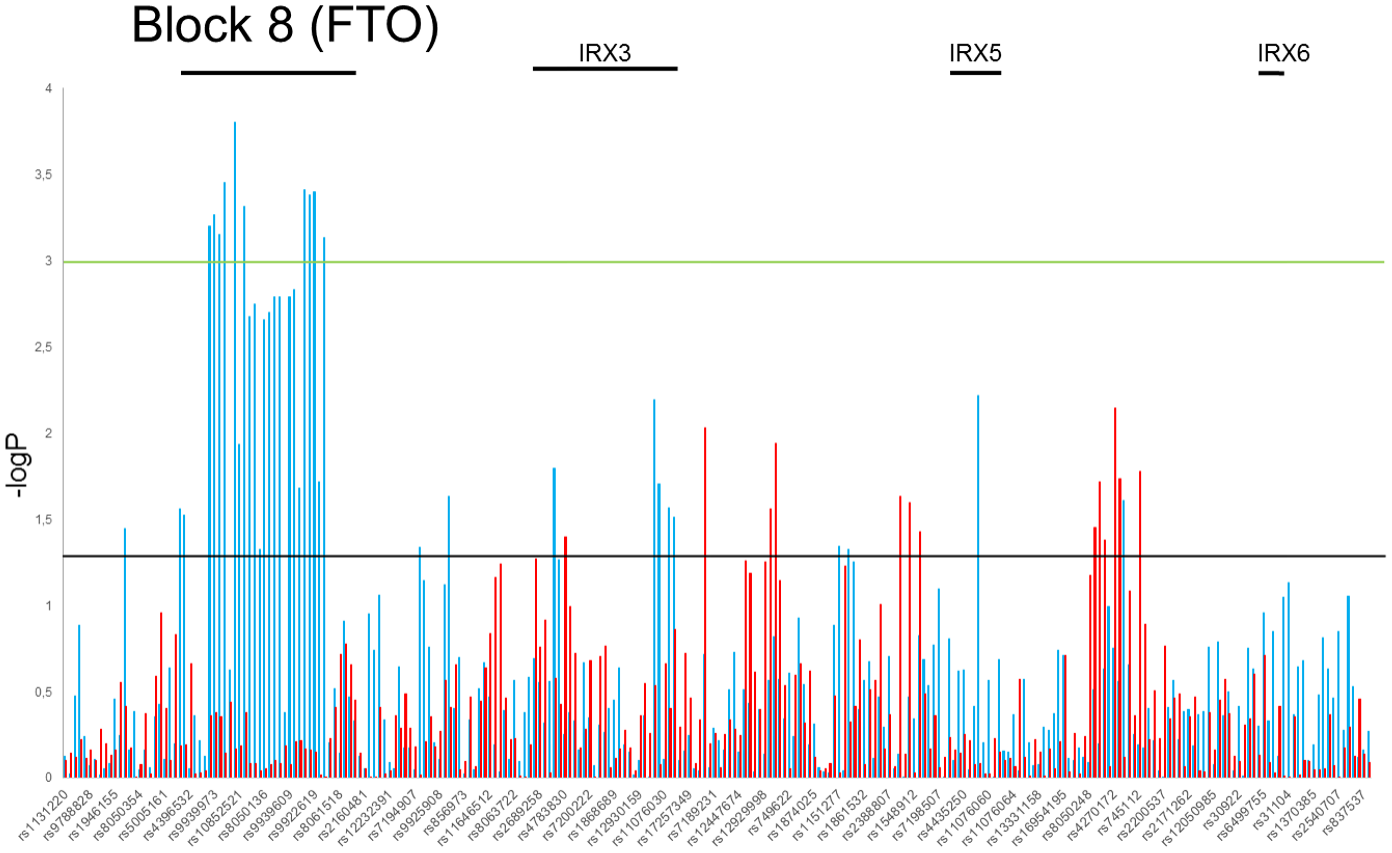
Distribution of estimated p values of the Chi square test in overweight phenotype stratified by age and sex. P values were calculated in the test of independence between the overweight phenotype and MAFs at tested SNPs (S5 Table). The *y* axis shows the −log_10_ p values of 262 SNPs obtained in overweight versus normal weight association analysis, and the *x* axis shows their rs numbers. Horizontal black and green lines represent the thresholds of p = 0.05 for significance without multiple correction and p = 8.2E-04 for LD block significance, respectively. Red and blue columns represents females and males group, respectively.

We have also performed analyses on different age groups in female and male. Six different age classes were set up in every 10 years interval (20–29, 30–39, 40–49, 50–59, 60–69, 70–79). Using CMH we tested the association of every SNP with overweight and/or obesity while assuming age class stratification. The observed effect of the SNPs impact on overweight and obesity was similar as described above but the estimated p values were lower while taking into account age stratification (S8 and S9 Tables). Differences in the distribution of estimated p values of the Chi square test between sexes in the normal weight versus obesity and normal weight and overweight association analysis with age stratification are depicted at Figs 3 and 4, respectively. This entirely confirmed the statistically significant association of block 8 SNPs with overweight group of male found in our analyses.

The *FTO* rs1558902 had the highest observed chi-square statistic (χ^2^ = 14.28, p = 0.0001572, OR = 1.265, SE = 0.06227, 95% CI = 1.12–1.43) in group of overweight men versus normal weight, whereas rs4270172, located 200 kb upstream *IRX3*, had the highest observed chi-square statistic (χ^2^ = 7.241, p = 0.007125, OR = 0.8063, SE = 0.08054, 95% CI = 0.6885–0.9442) in overweight females. When we analyzed the normal weight group versus group of obese women and obese men, the most statistically significant variants were located in *FTO*: rs1558902χ^2^ = 11.38, p = 0.000741, OR = 1.349, 95% CI = 1.133–1.605 and χ^2^ = 10.55, p = 0.001162, OR = 1.317, 95% CI = 1.115–1.556, respectively)) and rs12149832 (χ^2^ = 10.84, p = 0.000994, OR = 1.338, 95% CI 1.125–1.592 and χ^2^ = 10.09, p = 0.001493, OR = 1.309, 95% CI = 1.108–1.546, respectively).

We found additional association regions, in normal weight versus obesity analysis, which were located upstream *IRX3* (block 29 and 30, highest observed p value for rs1420285: χ^2^ = 10.84, p = 0.000994, OR = 1.338, 95% CI = 1.125–1.592) and downstream *IRX6* (blocks 53 and 54 with highest observed p value for rs2171262: χ^2^ = 10.87, p = 0.000975, OR = 1.431, 95% CI = 1.155–1.772) in females only (Fig 3). When we pulled together overweight and obesity groups we realized that in females group there are only 8 statistically significant SNPs compared with 27 in males (S6 Table).

We then searched for differences in the distributions of allele/genotype frequencies in case and control groups under additive, dominant, and recessive inheritance models. Stratification by BMI and sex revealed the influence of sex on the effect of tested SNPs on increased BMI in a genetic model-dependent manner (S10–S12 Tables). The strongest effect on obesity occurred again with *FTO* variants: rs1558902 and rs1421085 in females using a dominant model (p = 0.000463 and p = 0.0004744 respectively). In a group of males, two different *FTO loci*, rs12149832 and rs9930506, showed the strongest association with obesity under the recessive model (p = 0.003137 and 0.003741, respectively). Under the recessive inheritance model we also observed the strongest statistically significant association with BMI in range 25–29.99 kg/m^2^ for three *loci* in males and only one in females: rs4435250 (non-coding sequence located 100 kb downstream *IRX5*), rs9930506, rs12149832 (both *FTO* SNPs) and rs12444481 (non-coding sequence located 200 kb downstream *IRX5*), respectively.

**Fig 5:**
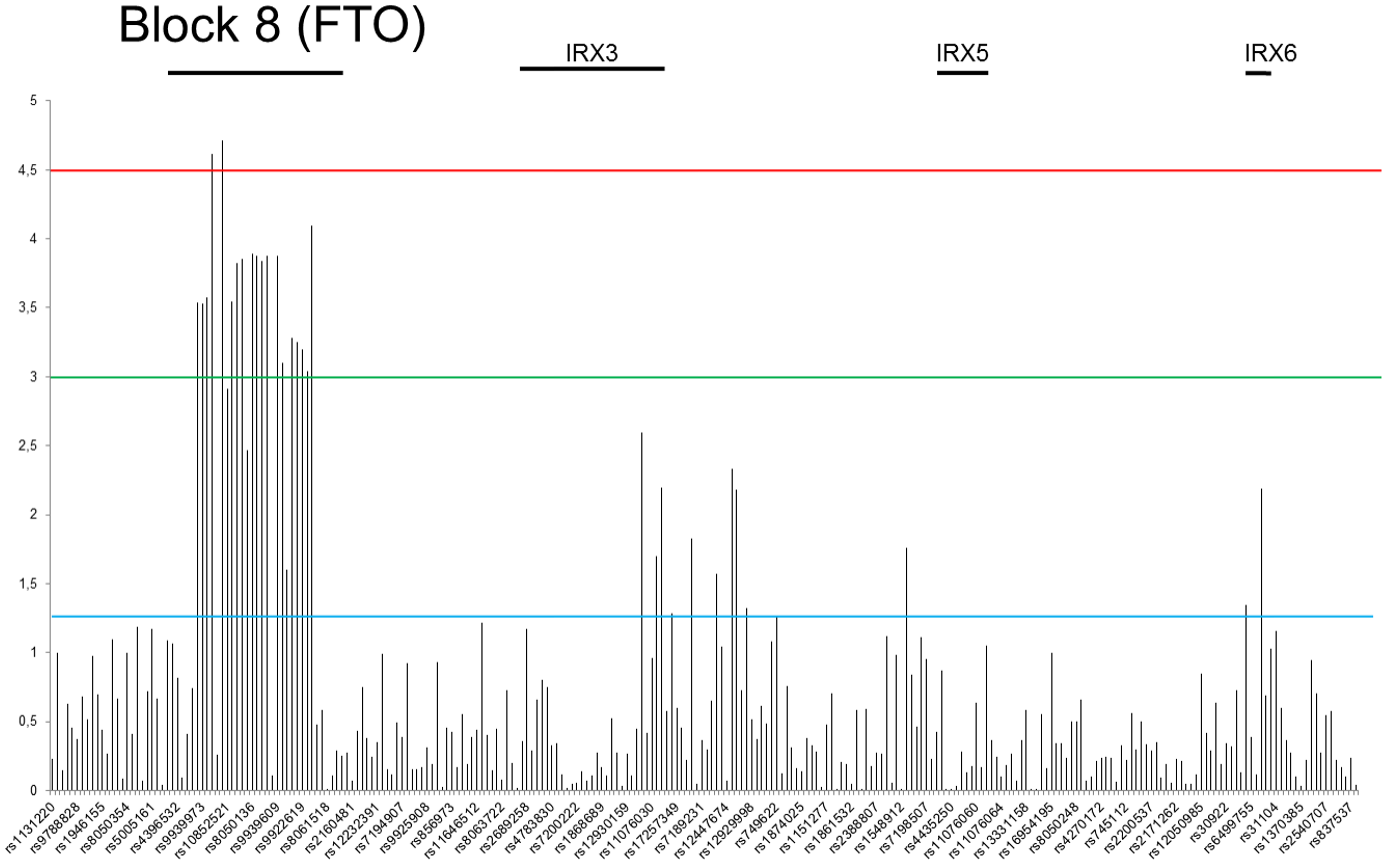
Distribution of calculated p values in linear regression analysis. P values are expressed as negative logarithm of the p values obtained in linear regression analysis with age and sex as a covariates. The *y* axis shows the −log_10_ p values of 262 SNPs, and the *x* axis shows their rs numbers. Horizontal blue, green and red lines represent the thresholds of p = 0.05 for significance without multiple correction, p = 8.2E-04 for LD block significance and p = 1.9E -04 for Bonferroni correction, respectively.

Previous studies suggested that genetic association to obesity at the *FTO locus* may be age dependent [40–42]. According to this, we examined the role of age in our age-diverse group (range from 20 to 77). Linear regression using BMI as a continuous trait (including underweight subjects) with sex and age as a covariates revealed differential contributions of SNPs to increased BMI. Obtained results are presented in Fig 5 and S13 Table. Age had bigger influence on BMI in group of women than in males. There were SNPs with positive and negative regression coefficient. The highest positive regression coefficient was observed only for three *FTO* variants: rs1558902, rs1421085 and rs9939609. The minor alleles of these SNPs were significantly more frequent in the obese group than in the normal-weight control group (0.5075 vs. 0.4509, 0.5069 vs. 0.4516 and 0.4812 vs. 0.4354, respectively) and carriers of the mentioned MAF had higher BMI than homozygous for the wild-type alleles (Beta = 0.3492, 95% CI = 0.1892–0.5093, p = 1.93E-05; Beta = 0.345, 95% CI = 0.185–0.5051, p = 2.42E-05; Beta = 0.3124, 95% CI = 0.1523–0.4725, p = 0.0001329, respectively).

## Discussion

In our study, we tested the genetic variation of 2 Mb region of chromosome 16, encompassing region of *FTO*, *RPGRIP1L*, *AKTIP*, *RBL2*, *IRX3*, *IRX5* and, *IRX6* genes. It was the first study with representation of individuals from each geographical region of Poland (N = 5418) focusing on this topic. Case—control association analysis was performed for 262 SNPs located in the tested region. Obtained results revealed that *FTO* SNPs rs1558902, rs1421085, rs12149832, rs9930506, rs9939609 had the strongest influence on obesity phenotype in our study group which is consistent with other studies [8–10, 27, 43–50]. These SNPs were in almost perfect LD (|D’|>0.98) with other 19 SNPs in block 8 located in non-coding sequence of intron 1. The haplotype analysis exhibited that the strongest obesity associated haplotype (χ^2^ = 11.474, p = 0.0007) which contained the minor alleles for SNPs distributed across a 45-kb stretch of intron 1 of *FTO*, was in block 8 and occurred at a frequency of 0.46 in the case group and 0.41 in controls. This region identified in our cohort was previously described by Hotta et al. as the 45 kb obesity associated region of high LD encompassing introns 1 and 2 of the *FTO* [48].

The most important discovery in our analysis was gender dependent manner of this association. SNPs in block 8 were statistically significant only in male group when we analyzed normal group versus overweight group. We did not observe this association in females. This unexpected finding was not observed so far in any previous studies. Thus, we concluded that overweight might be regulated by different genetic manner dependent on sex and in females is probably controlled by other region(s) in genome.

What is worth emphasizing, we have also noticed that SNPs included to block 8 were associated with obesity in normal versus obese group in females when assuming dominant inheritance model and in males when assuming recessive inheritance model. This genetic model-dependent sexual dimorphism for obesity susceptibility was also found in Mexicans but in different manner [51]. *FTO* SNPs were associated with obesity in recessive model and dominant model for females and males, respectively. Previous studies which concerned about different populations, reported that increased BMI heritability has sex-based differences and obesity trait is mediated through *FTO* and occurs independently in both sexes [51–53]. Meta-analysis of GWA studies of waist-hip ratio revealed evidence of gene-by-sex interactions involvement in body fat distribution [54]. Our study clearly demonstrates the importance of sex-stratification when analyzing complex traits. Sex can be perceived as risk factor which incorporates behavioral, physiological, and anatomical differences [55].

As the genetic association to obesity at the *FTO locus* was reported to be age dependent [40–42], we also examined the role of age in our age-diverse group (range from 20 to 77). Linear regression using BMI as a continuous trait and age and sex as a covariates revealed differential intronic *FTO* SNP contributions and disclosed that both age and sex had strong influence on BMI in our cohort (p = 3.06 × 10^−145^ and p = 9.05 × 10^−34^, respectively). We observed stronger association of intronic *FTO* region with BMI also when taking into account age class stratification in allelic association analysis.

The intronic region of *FTO* with high LD is packed with *cis*-regulatory elements, transcription factors binding sites, and DNase hypersensitive sites. These non-coding variants within *FTO* are connected with *IRX3*. In our Polish study group, we found one *locus* rs1126960, located in coding sequence of *IRX3* and associated with obesity in males only when assuming additive and dominant models. This SNP was also associated with overweight under the recessive model in non sex-stratified analysis. Recent evidence suggests that *FTO* variants directly affect adipocyte function through targeting *IRX3* and *IRX5*. Presence of the *FTO* risk haplotype in lean children was found to be associated with increased adipocyte-specific expression of *IRX3* and *IRX5*, whereas it was unaffected in a group of obese children. *IRX3* expression was elevated in adipose tissues and isolated adipocytes of lean compared to obese children and negatively correlated with BMI [30]. This new mechanism conferring genetic obesity risk whereby variants in *FTO* directly affect adipocyte function through the activation of *IRX3* and *IRX5* was previously discovered in lean adults by Claussnitzer et al. [56]. Although the functional interpretation of mechanism by which *FTO* variants interact with *IRX3* and *IRX5* remain clearly described, the genetic variation of IRXB cluster was not featured well. Hunt et al. sequenced the region of chromosome 16 encompassing the TAD area that includes the IRXB cluster in 284 Danish men [38]. Across tested area they identified 13373 simple (bi-allelic) variants, of which 12392 were SNPs and 981 were indels.

The haplotype analysis revealed that SNPs included in block 30, localized 200 kb upstream *IRX3*, were also associated with higher BMI (p = 0.0079). What is very interesting, we found that two SNPs, *FTO* rs1421084 and rs4238775 located downstream *IRX5*, in distance 1.3 Mb were in perfect LD. Near the location of *IRX3* and *IRX5*, we also detected association between BMI and eleven SNPs located in non-coding region of chromosome 16 between *IRX3* and *IRX5* (16: 54 359 850–54 780 370) when assuming linear regression with age as a covariate. Hunt and colleagues [38] observed similar region upstream *IRX5* which was independent of *FTO* but had, like *FTO*, an age dependent aspect, and its effect on BMI was similar.

Our next important finding is association between BMI and another haplotype (p = 0.0097) which contains SNPs from block 57, and localized 8 kb downstream from *IRX6*. These SNPs from 57 block (16: 55 326 804–55 349 837) were in high LD (|D’|>0.98) with SNPs located in coding region of *IRX6* and included to block 58.

Few limitations of this research must be considered. All participants are Caucasians, therefore obtained results may not be extendable to other groups. The mean age of the study group (50.16±13.55 for individuals with obesity, and 45.48±14.25 for individuals with overweight) suggests that hormonal decline might be partially responsible for observed associations. We were unable to include to our statistical analysis many of significant factors such as level of physical activity, nutrient intake, socioeconomic status or other environmental covariates that might influence results, as they were not included in questionnaires.

The p values presented in our study are not corrected for multiple testing. We did not aim in our study to discover new variants with genetic susceptibility to obesity. They have been previously identified by many of GWA studies in many populations. We mainly focused on characteristic of the Polish population in *FTO* and IRXB cluster regions and checked whether or not it is possible to observe any of sex specific differences in our sex stratified analyses. This sophisticated approach has enabled us to discover several novel and important findings described above.

The question of what significance threshold is appropriate for GWAS seems to be unresolved [57]. The usage of the GWA study correction (p<7.2E-08) in our particular study is overly as we only include to our analysis 2 Mb region of chromosome 16. A Bonferroni correction for 262 SNPs, would give a p value significance threshold of 1.9E-04. If we use the number of LD blocks (n = 61) identified by Haploview to estimate the corrected p value threshold, which becomes 0.05/61 = 8.2E-04, we would have considered only the *FTO* intron variants: rs1558902, rs1421085, rs12149832, rs9940128, rs9930333 and rs9939973 as the statistically significant corresponding to obesity in normal versus obese group without adjustment for sex. We have also controlled our statistical significance by performing 100 000 permutation tests in Haploview. Only two variants rs1558902 and rs1421085 passed permutation p value threshold (0.0185 and 0.0218, respectively). The rs12149832 showed suggestive evidence (p = 0.0675) of association with obesity in tested population of Poles after permutation tests (S14 and S15 Tables).

In conclusion, we have comprehensively analyzed the association of genetic variants and haplotypes located in the chromosomal FTO/IRXB region with increased and declined values of BMI in large and well characterized Polish cohort. This is the first study focusing on full characteristics of *FTO* and IRXB regions in the Polish population. Moreover, we performed comprehensive case-control analysis for different subgroups based on BMI including also significance of sex and age. Our greatest finding from this study was sex-dependent manner of *FTO* associations with obesity/overweight phenotype. Different variants of *FTO* were associated with BMI values in a group of women and men. We have also confirmed dependence of *FTO* intronic SNPs with other polymorphisms from the region of IRXB cluster. Our observations highlight the necessity, and challenge to reveal the genetic basis of obesity complex trait in population of Poles but also give a novel point of view for *FTO* genetic association with overweight and obesity as a more complex and not yet well-known issue. Thus, in the future we plan to extend our research of obesity and overweight phenotype by performing GWA study to further analyze these traits in Poles.

## Supporting information

S1 Fig Haplotype association analysis. (TIF)

S2 Fig Association of SNPs with obesity phenotype in group of female and male without age stratification. P values were calculated in the test of independence between the obesity phenotype and MAFs at tested SNPs (S4 Table). The *y* axis shows the −log_10_ p values of 262 SNPs obtained in obesity versus normal weight association analysis, and the *x* axis shows their rs numbers. Horizontal black and green lines represent the thresholds of p = 0.05 for significance without multiple correction and p = 8.2E-04 for LD block significance, respectively. Red and blue columns represents females and males group, respectively.
(TIF)

S3 Fig Association of SNPs with overweight phenotype in group of female and male without age stratification. P values were calculated in the test of independence between the overweight phenotype and MAFs at tested SNPs (S5 Table). The *y* axis shows the −log_10_ p values of 262 SNPs obtained in overweight versus normal weight association analysis, and the *x* axis shows their rs numbers. Horizontal black and green lines represent the thresholds of p = 0.05 for significance without multiple correction and p = 8.2E-04 for LD block significance, respectively. Red and blue columns represents females and males group, respectively.
(TIF)

S4 Fig Distribution of estimated p values of the Chi square test of obesity phenotype without sex and age stratification. P values were calculated in the test of independence between the obesity phenotype and MAFs at tested SNPs (S7 Table). The *y* axis shows the −log_10_ p values of 262 SNPs obtained in obesity versus normal weight association analysis, and the *x* axis shows their rs numbers. Horizontal black and green lines represent the thresholds of p = 0.05 for significance without multiple correction and p = 8.2E-04 for LD block significance, respectively. Red and blue columns represents females and males group, respectively.
(TIF)

S1 Table Minor allele frequencies (MAF) for tested SNPs. The threshold was set up on MAF<0.01.
(XLSX)

S2 Table Genotype frequencies of all the cohort, case and control groups and calculated Hardy-Weinberg test statistics (HWE). SNP—SNP identifier, Group—Code indicating sample (ALL-all cohort, Case-obesity, Control-normal weight), A—Minor allele code, B—Major allele code, AA/AB/BB—Genotype counts, O(HET), E(HET)—Observed and Expected heterozygosity, respectively; p—HWE p-value. The cut off was set up (p<1E-04).
(XLSX)

S3 Table Haplotype association with obesity in study group. Bold font denotes haplotypes with statistically significant p values.
(XLSX)

S4 Table Case-control association analysis of 262 SNPs from tested region with obesity (BMI ≥ 30 kg/m^2^), stratified by sex. SNP—rs number of tested SNP; BP—Physical position of tested SNP on chromosome 16; A, B—Minor and major allele names, respectively (based on whole sample); F_A—Frequency of MAF in cases; F_U—Frequency of MAF allele in controls; CHISQ—Basic allelic test chi-square (1df); P—Asymptotic p-value for this test; OR—Estimated odds ratio (for A, i.e. B is reference); L95—Lower bound of 95% confidence interval for odds ratio; U95—Upper bound of 95% confidence interval for odds ratio. Bold font denotes markers with statistically significant p values.
(XLSX)

S5 Table Case-control association analysis of 262 SNPs from tested region with overweight (BMI 25–29.99 kg/m^2^), stratified by sex. SNP—rs number of tested SNP; BP—Physical position of tested SNP on chromosome 16; A, B—Minor and major allele names, respectively (based on whole sample); F_A—Frequency of MAF in cases; F_U—Frequency of MAF allele in controls; CHISQ—Basic allelic test chi-square (1df); P—Asymptotic p-value for this test; OR—Estimated odds ratio (for A, i.e. B is reference); L95—Lower bound of 95% confidence interval for odds ratio; U95—Upper bound of 95% confidence interval for odds ratio. Bold font denotes markers with statistically significant p values.
(XLSX)

S6 Table Case-control association of 262 SNPs from tested region with overweight and obesity (BMI ≥25 kg/m^2^), stratified by sex. SNP—rs number of tested SNP; BP—Physical position of tested SNP on chromosome 16; A, B—Minor and major allele names, respectively (based on whole sample); F_A—Frequency of MAF in cases; F_U—Frequency of MAF allele in controls; CHISQ—Basic allelic test chi-square (1df); P—Asymptotic p-value for this test; OR—Estimated odds ratio (for A, i.e. B is reference); L95—Lower bound of 95% confidence interval for odds ratio; U95—Upper bound of 95% confidence interval for odds ratio. Bold font denotes markers with statistically significant p values.
(XLSX)

S7 Table Case-control association of 262 SNPs from tested region with obesity (BMI ≥30 kg/m^2^), without sex stratification. SNP—rs number of tested SNP; BP—Physical position of tested SNP on chromosome 16; A, B—Minor and major allele names, respectively (based on whole sample); F_A—Frequency of MAF in cases; F_U—Frequency of MAF allele in controls; CHISQ—Basic allelic test chi-square (1df); P—Asymptotic p-value for this test; OR—Estimated odds ratio (for A, i.e. B is reference); L95—Lower bound of 95% confidence interval for odds ratio; U95—Upper bound of 95% confidence interval for odds ratio. Bold font denotes markers with statistically significant p values.
(XLSX)

S8 Table Case-control association of 262 SNPs from tested region with obesity (BMI ≥30 kg/m^2^), stratified by sex and age class. SNP—rs number of tested SNP; BP—Physical position of tested SNP on chromosome 16; A, B—Minor and major allele names, respectively (based on whole sample); F_A—Frequency of MAF in cases; F_U—Frequency of MAF allele in controls; CHISQ—Basic allelic test chi-square (1df); P—Asymptotic p-value for this test; OR—Estimated odds ratio (for A, i.e. B is reference); L95—Lower bound of 95% confidence interval for odds ratio; U95—Upper bound of 95% confidence interval for odds ratio. Bold font denotes markers with statistically significant p values.
(XLSX)

S9 Table Case-control association of 262 SNPs from tested region with overweight (BMI 25–29.99 kg/m^2^), stratified by sex and age class. SNP—rs number of tested SNP; BP—Physical position of tested SNP on chromosome 16; A, B—Minor and major allele names, respectively (based on whole sample); F_A—Frequency of MAF in cases; F_U—Frequency of MAF allele in controls; CHISQ—Basic allelic test chi-square (1df); P—Asymptotic p-value for this test; OR—Estimated odds ratio (for A, i.e. B is reference); L95—Lower bound of 95% confidence interval for odds ratio; U95—Upper bound of 95% confidence interval for odds ratio. Bold font denotes markers with statistically significant p values.
(XLSX)

S10 Table Full model association of tested SNPs with obesity in Polish population. AFF—Genotypes/alleles in cases, UNAFF—Genotypes/alleles in controls, GENO—Genotypic model (AA versus AB versus BB); ADD—Additive model (AA versus BB); DOM—Dominant model (AA+AB versus BB); REC—Recessive model (AA versus AB+BB); CHISQ—Chi-squated statistic, P—Asymptotic p-value. By convention, in all models, A is the minor allele and B is the major allele. Bold font denotes markers with statistically significant p values.
(XLSX)

S11 Table Full model association of tested SNPs with overweight. AFF—Genotypes/alleles in cases, UNAFF—Genotypes/alleles in controls, GENO—Genotypic model (AA versus AB versus BB); ADD—Additive model (AA versus BB); DOM—Dominant model (AA+AB versus BB); REC—Recessive model (AA versus AB+BB); CHISQ—Chi-squated statistic, P—Asymptotic p-value. By convention, in all models, A is the minor allele and B is the major allele. Bold font denotes markers with statistically significant p values.
(XLSX)

S12 Table Full model association of tested SNPs with overweight and obesity. AFF—Genotypes/alleles in cases, UNAFF—Genotypes/alleles in controls, GENO—Genotypic model (AA versus AB versus BB); ADD—Additive model (AA versus BB); DOM—Dominant model (AA+AB versus BB); REC—Recessive model (AA versus AB+BB); CHISQ—Chi-squated statistic, P—Asymptotic p-value. By convention, in all models, A is the minor allele and B is the major allele. Bold font denotes markers with statistically significant p values.
(XLSX)

S13 Table Linear regression analysis of 262 SNPs in Polish population, adjusted for sex and age under additive model. SNP—rs number of tested SNP; BP—Physical position of SNP on chromosome 16; A—minor allele; NMISS—Number of non-missing individuals included in analysis; BETA—regression coefficient; SE—standard error of regression coefficient; L95—Lower bound of 95% confidence interval; U95—Upper bound of 95% confidence interval; STAT—Coefficient t-statistic; P—Asymptotic p-value for t-statistic, bold font denotes markers with statistically significant p values and shaded cells indicate the meaning the additive effects of allele dosage when assuming age and sex as a covariates.
(XLSX)

S14 Table Permutation analysis (n = 100000) performed for single markers. 1850 permutation exceed highest observed Chi-square (29.95). Best observed Chi-square 15.355 for rs1558902. Bold font denotes markers with statistically significant p values.
(XLSX)

S15 Table Permutation analysis (n = 100000) performed for haplotypes. 15527 permutation exceed highest observed Chi-square (29.035). Best observed Chi-square 11.474 for Block 8. No statistically significant association.
(XLSX)
