# Supplementary figures and images for: Genetic association of FTO/IRX region with obesity and overweight in the Polish population
Source: PLoS One. 2017 Jun 29;12(6):e0180295. doi: 10.1371/journal.pone.0180295 (PMC5491248; doi:10.1371/journal.pone.0180295)

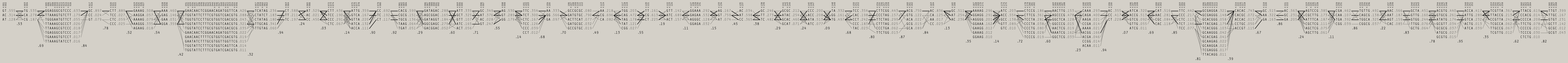

Supplement: S1 Fig — (TIF) [file pone.0180295.s001.TIF]

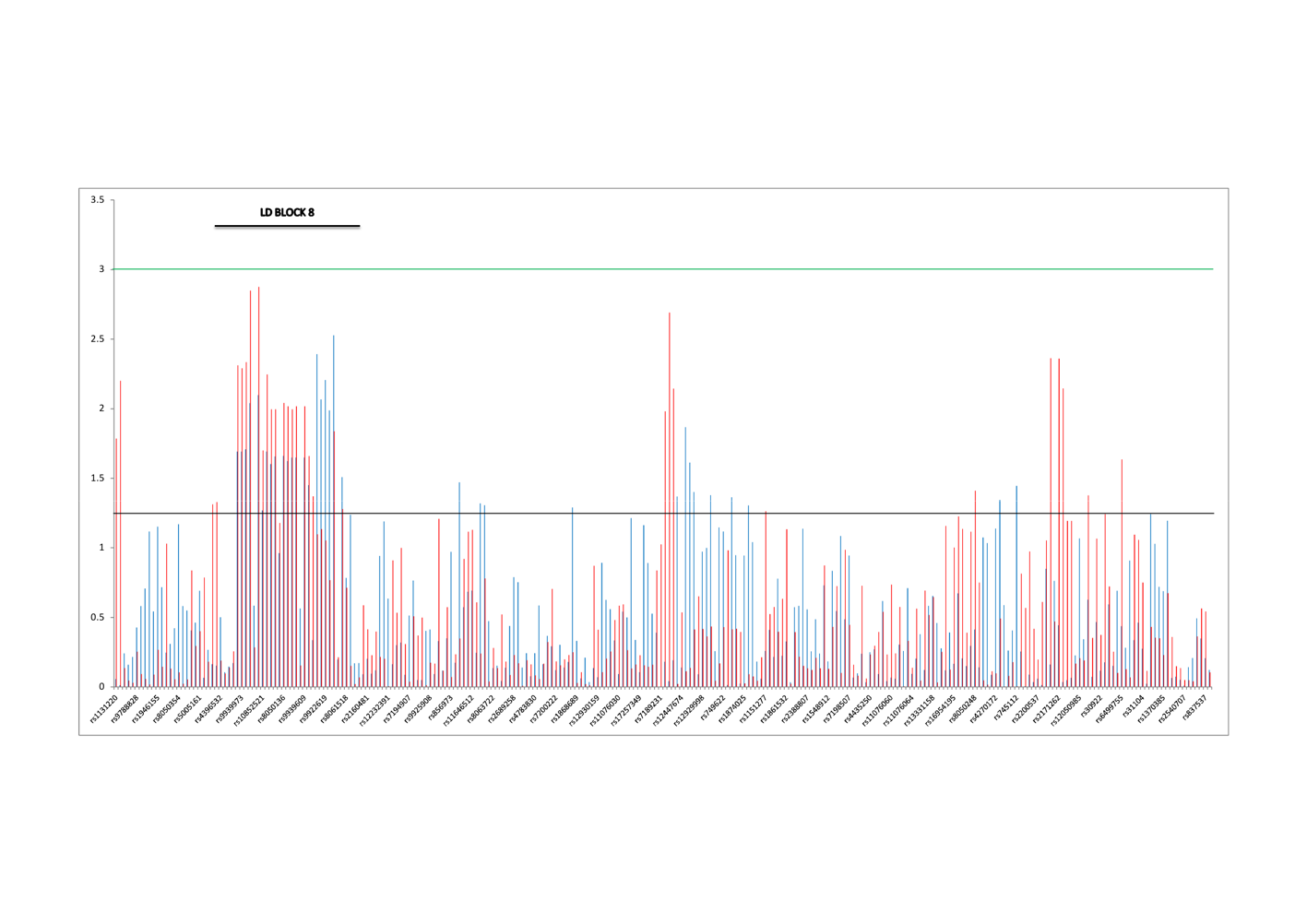

Supplement: S2 Fig — P values were calculated in the test of independence between the obesity phenotype and MAFs at tested SNPs (S4 Table). The y axis shows the −log10 p values of 262 SNPs obtained in obesity versus normal weight association analysis, and the x axis shows their rs numbers. Horizontal black and green lines represent the thresholds of p = 0.05 for significance without multiple correction and p = 8.2E-04 for LD block significance, respectively. Red and blue columns represents females and males group, respectively. (TIF) [file pone.0180295.s002.TIF]

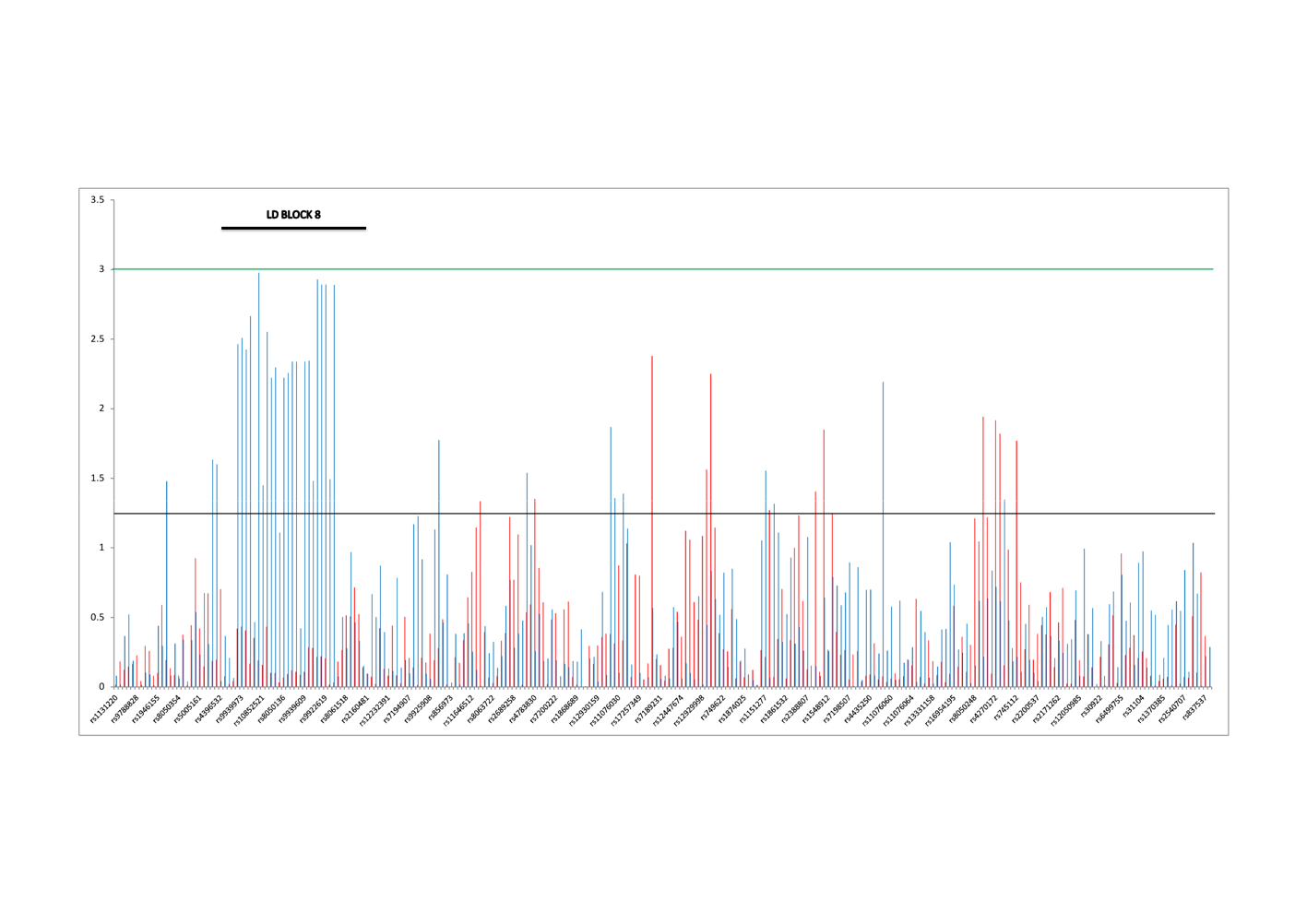

Supplement: S3 Fig — P values were calculated in the test of independence between the overweight phenotype and MAFs at tested SNPs (S5 Table). The y axis shows the −log10 p values of 262 SNPs obtained in overweight versus normal weight association analysis, and the x axis shows their rs numbers. Horizontal black and green lines represent the thresholds of p = 0.05 for significance without multiple correction and p = 8.2E-04 for LD block significance, respectively. Red and blue columns represents females and males group, respectively. (TIF) [file pone.0180295.s003.tif]

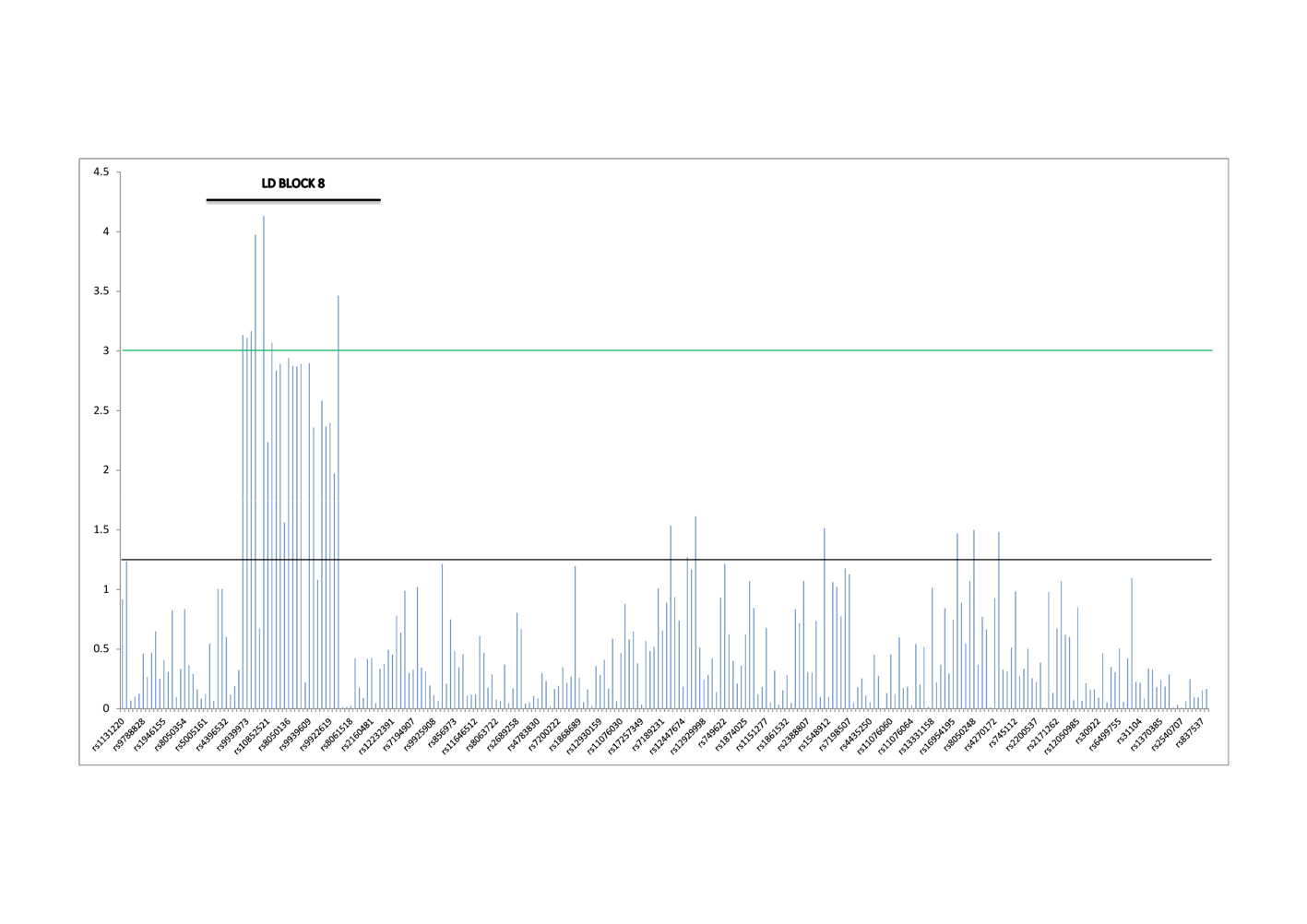

Supplement: S4 Fig — P values were calculated in the test of independence between the obesity phenotype and MAFs at tested SNPs (S7 Table). The y axis shows the −log10 p values of 262 SNPs obtained in obesity versus normal weight association analysis, and the x axis shows their rs numbers. Horizontal black and green lines represent the thresholds of p = 0.05 for significance without multiple correction and p = 8.2E-04 for LD block significance, respectively. Red and blue columns represents females and males group, respectively. (TIF) [file pone.0180295.s004.tif]
